# Surgical treatment and prognosis of gastric neuroendocrine neoplasms: a single-center experience

**DOI:** 10.1186/s12876-016-0505-5

**Published:** 2016-09-09

**Authors:** Chaoyong Shen, Huijiao Chen, Haining Chen, Yuan Yin, Luyin Han, Jiaju Chen, Sumin Tang, Xiaonan Yin, Zongguang Zhou, Bo Zhang, Zhixin Chen

**Affiliations:** 1 Department of Gastrointestinal Surgery, West China Hospital, Sichuan University, Chengdu, 610041 Sichuan China; 2 Department of Pathology, West China Hospital, Sichuan University, Chengdu, 610041 Sichuan China; 3 Intensive Care Unit, West China Hospital, Sichuan University, Chengdu, 610041 China; 4 Institute of Digestive Surgery and State key Laboratory of Biotherapy, West China Hospital, Sichuan University, Chengdu, 610041 Sichuan China

**Keywords:** Neuroendocrine neoplasms, New WHO grading, Prognosis, Surgery, Stomach

## Abstract

**Background:**

Gastric neuroendocrine neoplasms (G-NENs) are uncommon, and data on their management is limited. We here investigated the clinicopathological characteristics, surgical and survival outcomes in G-NENs among Chinese. Moreover, we will discuss their prognostic value.

**Methods:**

From existing databases of the West China Hospital, we retrospectively identified 135 consecutive patients who were surgically treated and pathologically diagnosed as G-NENs from January 2009 to August 2015.

**Results:**

This entire cohort comprised 98 males and 37 females, with a median age of 60 years. Twenty-five patients underwent endoscopic resection, while 110 patients underwent open/laparoscopic surgery. Thirty-nine patients had neuroendocrine tumor G1 (NET G1), seven patients had neuroendocrine tumor G2 (NET G2), 69 patients had neuroendocrine carcinoma G3 (NEC G3) and 20 patients had mixed adenoneuroendocrine carcinoma (MANEC). The median survival was not achieved for both NET G1 and NET G2 versus 19 months (range 3–48) for NEC G3 and 10.5 months (range 3–45) for MANEC. The 3-year survival rates for stage I, II, III, and IV were 91.1 %, 78.6 %, 51.1 % and 11.8 %, respectively (*P* < 0.001). As for the prognostic analysis, both surgical margin and the newly updated World Health Organization (WHO) classification were independent predictors of overall survival (OS).

**Conclusions:**

G-NENs are a kind of rare tumors, and patients with NET G3 and MANEC have unfavorable prognosis even surgically treated. Moreover, surgical margin and the new 2010 WHO criteria are closely associated with OS for G-NENs.

## Background

Gastric neuroendocrine neoplasms (G-NENs) are a heterogeneous group of neoplasms, showing different clinicopathological characteristics and behaviors, and it composed of cells containing neuroendocrine secretory granules in their cytoplasm [1, 2]. G-NENs, with an incidence of 0.3 per 100000 each year, are uncommon, accounting for only 4 % of all NENs of the body [3, 4]. The incidence of G-NENs has increased dramatically over the last decades, which may attributed to factors such as increased clinical and pathological experience in diagnosing this disease, as well as heightened physician awareness and increased endoscopic surveillance. Nowadays, surgical resection undoubtedly remains the mainstay of the potentially curative treatment for the G-NENs. Thus, various types of surgical approaches have already been performed for G-NENs, such as traditional open surgery, endoscopic and laparoscopic resection [5, 6]. Moreover, G-NENs often have unpredictable biological behaviors. Some are associated with a very aggressive clinical course, despite they are clinically silent [7, 8]. Thus, treating G-NENs is often a challenge for the attending clinicians.

The proper management of NENs is the ability to stratify patients into prognostic groups. Nevertheless, the prognostic classification of G-NENs has been challenging due to their rarity. The world Health Organization (WHO) in 2000 classified G-NENs into 3 categories: well differentiated neuroendocrine tumor, well-differentiated neuroendocrine carcinoma, and poorly differentiated neuroendocrine carcinoma [9]. Successively, the European Neuroendocrine Tumor Society (ENETS) in 2006 and the Union for International Cancer Control and the American Joint Cancer Committee (UICC/AJCC) in 2010 proposed the tumor-node-metastasis (TNM) staging system for G-NENs, respectively [10, 11]. The ENETs TNM staging seems work better in the prognostic stratification for pancreas, while the UICC/AJCC might be superior to ENENTs for appendix [12, 13]. In 2010, for wider acceptance, the WHO updated its classification system into four different categories: neuroendocrine tumor G1 (NET G1), neuroendocrine tumor G2 (NET G2), neuroendocrine carcinoma G3 (NEC G3) and mixed adenoneuroendocrine carcinoma (MANEC) [14]. However, the studies have specifically evaluated G-NENs using this new classification are rare till now.

To the best of our knowledge, studies on evaluation of clinicopathological characteristics, surgical outcome and prognosis for G-NENs with such a large sample in a single institution are uncommon due to their rarity and heterogeneity, especially using the newly 2010 WHO classification. Therefore, we here aimed to explore the clinicopathological characteristics and surgical outcomes on the basis of 135 consecutive patients with G-NENs at our center, as well as to evaluate the prognostic value of the new WHO criteria.

## Methods

### Patient selection

The medical records of all consecutive patients with G-NENs were retrospectively obtained from the West China Hospital, Sichuan University between January 2009 and August 2015. The Institutional Review Board and Ethics Committee of the West China Hospital of Sichuan University deemed that an ethical review was not needed for this retrospective analysis. Written informed consent was obtained from all patients. The inclusion criteria were as follows: (1) Patients were surgically treated and pathologically diagnosed as having primary G-NENs by the pathologists at our institution. (2) The pathological diagnosis of the G-NENs considered the typical morphological findings and the expression of neuroendocrine markers, such as Chromogranin A and/or Synaptophysin and so on (the partial expression of several neuroendocrine markers was considered as negative expression). The exclusion criteria were as follows: (1) Patients with G-NENs synchronous with other malignancies and insufficient medical charts were excluded. (2) Patients received only radiofrequency thermal ablation and/or transarterial embolization and/or transarterial chemoembolization due to extensive distant metastasis. (3) Adenocarcinomas with scattered neuroendocrine cells or with a focal neuroendocrine component cannot be considered as MANEC were excluded. MANEC diagnosis was confirmed when the characteristics of coexistence of exocrine and neuroendocrine components were identified, with each of them accounting for at least 30 % of the lesion [14]. TNM staging was evaluated according to the guidelines published by the AJCC [11]. The tumors were graded as NET G1, NET G2, NEC G3, and MANEC following the newly 2010 WHO classification. And the definition of each grade is as follows: NET G1: mitotic count < 2/10 high power fields (HPF) and/or Ki-67 index ≤2 %; NET G2: mitotic count 2–20/10 HPF and/or Ki-67 index 3–20 %; NEC G3: mitotic count >20/10 HPF and/or Ki-67 index >20 % [14].

### Surgery and medical treatment

Patients with G-NENs underwent surgical treatment with curative intent. The surgical procedures included endoscopic, laparoscopic resection and traditional open surgery. Computerized tomography (CT) and/or endoscopic ultrasonography were performed in all cases preoperatively to determine tumor location and size, depth of invasion, local lymphatic metastasis and distant metastasis. All endoscopic resections were performed by skilled endoscopic specialists and surgeons. Endoscopic complete resection is regarded as the absence of residual tumor tissue macroscopically on endoscopy and microscopically. Patients treated with open or laparoscopic resection underwent subtotal, total gastrectomy or wedge resection. The lesions were preoperatively diagnosed as the possibility of benign and/or low-grade malignant gastric NENs, and without any evidence of distant metastasis and a history of epigastrium surgery, had undergone laparoscopic surgery. Furthermore, multivisceral resection was performed for tumors that invaded adjacent tissues and organs. Frozen slices of incisal margin and surgical specimen were routinely collected during surgery. The surgery was classified into three categories: R0 (complete gross and microscopic resection), R1 (microscopic residual lesions), and R2 resections (the presence of any gross residua tumors). Adjuvant chemotherapy was recommended for patients with metastatic G-NENs, R1/R2 resection and NEC/MANEC.

### Data collection and follow-up

The parameters that were retrospectively reviewed from their medical charts included age at diagnosis, gender, location of primary tumor, immunohistochemical staining, tumor markers, co-morbidity (including diabetes mellitus, chronic pulmonary disease, cardiovascular, chronic liver and renal disease), neutrophil-lymphocyte ratio (NLR), albumin/globulin ratio (A/G ratio), tumor grade, vascular invasion, tumor TNM stage at diagnosis, type of surgery, surgical outcome, and survival outcome, etc. Follow-up was conducted by office visit, telephone call, or outpatient clinic visit from October 2015 to November 2015. Abdominal CT and/or endoscopic ultrasonography, blood routine examination, and evaluation of liver and kidney functions were also performed.

### Survival and statistical analysis

Overall survival (OS) was defined as the time from the start of treatment until death from any cause or last follow-up visit. Measurement data were expressed as mean ± standard deviation. Differences among groups were analyzed using analysis of variance for continuous variables and χ^2^ test or Fisher’s exact test for categorical data. Wilcoxon test was used to test ranked data. OS was calculated using the Kaplan-Meier method and compared using a log-rank test. Multivariate analyses using the Cox proportional hazards model were carried out to identify factors independently associated with prognosis. Differences with two-sided *P* < 0.05 indicated statistical significance. All statistical analyses were performed using Statistical Package for Social Science (SPSS Inc., Chicago, IL, USA).

## Results

### Clinicopathological and demographic characteristics

The demographic and clinical characteristics are summarized in Table 1. In total, one hundred thirty-five patients with G-NENs were identified. This entire cohort comprised 98 males and 37 females, with a male-to-female ration of 2.6. Median age at initial diagnosis was 60 years (range 28–81), with a mean of 58.2 ± 12.0 years. In patients reporting main symptoms upon initial presentation, 84 patients exhibited abdominal pain/discomfort, 21 patients with dysphagia, 6 patients with gastrointestinal bleeding, 5 patients with heartburn, 1 patient with abdominal mass, and 18 patients were asymptomatic. Tumor diameters ranged from 0.3 to 14 cm and with a median of 4.0 cm, with an average of 3.8 ± 2.6 cm. There were 39, 7, 69, and 20 patients had NET G1, NET G2, NEC G3 and MANEC, respectively. The NEC G3 included small cell and large cell type in 40 and 29 patients, respectively. No statistical significances were observed with respect to age, gender, lesion diameter, tumor location, depth of invasion, lymph node metastasis, vascular invasion and TNM stage between small cell and large cell type (*P* > 0.05). In addition, there were significantly more males than females with NET G3 and MANEC comparison to NET G1 (*P* = 0.001, *P* < 0.001, respectively). NET G1 and NET G2 had smaller tumor size than that of NEC G3 and MANEC (NET G1 vs NEC G3, *P* < 0.001; NET G1 vs MANEC, *P* < 0.001; NET G2 vs NEC G3, *P* < 0.001; NET G2 vs MANEC, *P* = 0.005), but no significant difference was noted between NET G1 and NET G2 (*P* = 0.115). There were more tumors exhibited mucosal ulcer in NET G2, NEC G3 and MANEC than that of NET G1 (*P* < 0.001). Furthermore, there were statistical significances with respect to age, co-morbidity, hospital stay and tumor location among these four groups (*P* <0.05). However, the number of lesions, hemoglobin level, NLR, A/G ratio, and tumor markers did not differ among the four groups.

**Table 1:** Demographic and clinical characteristics of the patients with G-NENs (*n* = 135)

Variables	NET G1 (*n* = 39, %)	NET G2 (*n* = 7, %)	NEC G3 (*n* = 69, %)	MANEC (*n* = 20, %)	*P* value
Gender					<0.001
Male	19 (48.7)	5 (71.4)	54 (78.3)	20 (100.0)	
Female	20 (51.3)	2 (28.6)	15 (21.7)	0 (0.0)	
Age, y	53.3 ± 13.0	47.0 ± 13.1	61.0 ± 10.3	62.2 ± 10.5	<0.001
Lesion diameter, cm	1.2 ± 0.9	2.4 ± 1.8	5.2 ± 1.8	4.7 ± 3.1	<0.001
Hospital stay, days	10.7 ± 4.9	14.9 ± 5.6	16.0 ± 5.0	17.1 ± 5.5	<0.001
Tumor location					<0.001
U	7 (17.9)	2 (28.6)	36 (52.2)	9 (45.0)	
M	29 (74.4)	3 (42.9)	11 (15.9)	2 (10.0)	
L	3 (7.7)	2 (28.6)	22 (31.9)	9 (45.0)	
Number of lesions					0.245
Multiple	8 (20.5)	1 (14.3)	1 (1.4)	0 (0.0)	
single	31 (79.5)	6 (85.7)	68 (98.6)	20 (100.0)	
Mucosal ulcer					<0.001
Present	10 (25.6)	7 (100.0)	68 (98.6)	19 (95.0)	
Absent	29 (74.4)	0 (0.0)	1 (1.4)	1 (5.0)	
Co-morbidity*					0.042
Present	5 (12.8)	2 (28.6)	14 (20.3)	9 (45.0)	
Absent	34 (87.2)	5 (71.4)	55 (79.7)	11 (55.0)	
Anemia					0.643
Yes	5 (12.8)	2 (28.6)	9 (13.0)	3 (15.0)	
No	34 (87.2)	5 (71.4)	60 (87.0)	17 (85.0)	
Hemoglobin level, g/L	127.8 ± 20.5	113.0 ± 20.6	124.1 ± 25.5	126.0 ± 20.3	0.463
NLR	2.5 ± 1.5	2.3 ± 1.6	3.0 ± 1.2	3.1 ± 2.1	0.197
A/G ratio	1.5 ± 0.3	1.5 ± 0.2	1.5 ± 0.3	1.6 ± 0.3	0.581
Tumor markers					
CEA↑	2 (5.1)	0 (0.0)	13 (18.8)	3 (15.0)	0.194
AFP↑	0 (0.0)	0 (0.0)	3 (4.3)	0 (0.0)	0.638
CA19-9↑	1 (2.6)	0 (0.0)	8 (11.6)	2 (10.0)	0.354
CA125↑	1 (2.6)	0 (0.0)	2 (2.9)	0 (0.0)	1.000
CA72-4↑	0 (0.0)	0 (0.0)	2 (2.9)	2 (10.0)	0.217

*G-NENs* gastric neuroendocrine neoplasms, *NET G1* neuroendocrine tumor G1, *NET G2* neuroendocrine tumor G2, *NEC G3* neuroendocrine carcinoma G3, *MANEC* mixed adenoneuroendocrine carcinoma, *U* upper third of stomach, *M* middle third of stomach, *L* lower third of stomach; Co-morbidity* including diabetes mellitus, chronic pulmonary disease, cardiovascular, chronic liver and renal disease; *NLR* Neutrophil-Lymphocyte Ratio, *A/G ratio* albumin/globulin ratio, *CEA* carcinoembryonic antigen, *AFP* alpha-fetoprotein, *CA19-9* carbohydrate antigen 19-9, *CA125* cancer antigen 125, *CA72-4* cancer antigen 72-4

### Surgical information and medical treatment

All patients were surgically treated in our institution, and details can be seen in Table 2. Twenty-five patients underwent endoscopic resection (ER), while 110 patients underwent open/laparoscopic surgery (3 cases with laparoscopic resection). Patients who underwent ER had a smaller tumor size than those with open/laparoscopic surgery (0.7 ± 0.4 cm vs 4.5 ± 2.3 cm *P* < 0.001). The depth of invasion was mostly T1 for ER (24/25 vs 15/110 for open/laparoscopic surgery). A total of 3 patients underwent multivisceral resection, consisting of 2 patients with Gastrectomy + splenectomy, and 1 patient with Gastrectomy + partial hepatectomy. One hundred fourteen patients underwent R0 resection, and 21 patients had R1/R2 resection (1 and 20 underwent endoscopic and open/laparoscopic resection, respectively). Fourteen patients experienced postoperative complications, such as abdominal infection (*n* = 2), intestinal obstruction (*n* = 3), alimentary tract bleeding (*n* = 5), and pulmonary infection (*n* = 4). No perioperative death occurred. A total of 10 patients received somatostatin analogues, while 20 patients (MANEC in 7 cases, NEC G3 in 11 cases) received cytotoxic chemotherapy. The most common chemotherapy combination regimens used as first-line therapy included etoposide-cisplatin (EP regimen, 9 patients), and irinotecan-cisplatin (IP regimen, 7 patients).

**Table 2:** Surgical and medical treatments used for G-NENs

Variables	Mean ± SD (Numbers/Percentage)
Surgical approaches	
Endoscopic resection	25/135 (18.5 %)
Open/Laparoscopic surgery	110/135 (81.5 %)
Types of gastrectomy*	
Proximal gastrectomy	38/110 (34.5 %)
Distal gastrectomy	39/110 (35.5 %)
Total gastrectomy	31/110 (28.2 %)
Wedge resection	2/110 (1.8)
Multivisceral resection#	
Gastrectomy + splenectomy	2/110 (1.8 %)
Gastrectomy + partial hepatectomy	1/110 (0.9 %)
Surgical margins	
R0	114/135 (84.4 %)
R1/R2	21/135 (15.6 %)
Surgical complications	
Abdominal infection	2/135 (1.5 %)
Intestinal obstruction	3/135 (2.2 %)
Alimentary tract bleeding	5/135 (3.7 %)
Pulmonary infection	4/135 (3.0 %)
Perioperative death	0/135 (0.0 %)
Postoperative medical treatments	
Cytotoxic chemotherapy	20 (14.8 %)
Somatostatin analogs	10 (7.4 %)

*G-NENs* gastric neuroendocrine neoplasms; *#including the patients who underwent Open/Laparoscopic surgery

### TNM stages and immunohistochemical characteristics

A TNM stage was assigned for each patient according to the UICC/AJCC S*taging Manual* (seventh edition in 2010). Chest and abdominal CT, as well as endoscopic ultrasonography were preoperatively performed to evaluate the depth of invasion and lymph node for patients who underwent endoscopic resection. There were 37, 16, 34, and 48 patients from stages T1 to T4, respectively. A total of 89 patients were pathologically confirmed to have invasion of lymph node, and the number of positive lymph node range from 1 to 42, with a median of 4. Seventeen patients had distant metastases, and the liver (*n* = 9, 52.9 %) was the most common site of distant metastasis. NET G1 was mostly in stage I/II (92.3 %); but those with NET G2, NEC G3 and MANEC were mainly in stage III/IV (57.1 %, 94.2 %, and 90.0 %, respectively). The NET G1 had less patients with vascular invasion than that of NET G2, and NEC G3 and MANEC (*P* = 0.032, *P* < 0.001, and *P* < 0.001, respectively). With regarding to depth of invasion, lymph node metastasis, and distant metastasis, significant differences were noted among four groups (*P* ≤ 0.001). A total of 108 patients (80.0 %) stained positive for Chromogranin A, 100 patients (74.1 %) for Synaptophysin, 29 patients (21.5 %) for Neuron Specific Enolase, and 63 patients (46.7 %) for CD56. However, there were no significant differences among four groups with respect to these immunohistochemical markers (*P* > 0.05). Details were listed in Table 3.

**Table 3:** Pathological and immunohistochemical characteristics in patients with G-NENs (*n* = 135)

Variables	NET G1 (*n* = 39, %)	NET G2 (*n* = 7, %)	NEC G3 (*n* = 69, %)	MANEC (*n* = 20, %)	*P* value
Depth of invasion					<0.001
T1	29 (74.4)	3 (42.9)	4 (5.8)	1 (5.0)	
T2	7 (17.9)	0 (0.0)	6 (8.7)	3 (15.0)	
T3	2 (5.1)	0 (0.0)	23 (33.3)	9 (45.0)	
T4	1 (2.6)	4 (57.1)	36 (52.2)	7 (35.0)	
Lymph node metastasis					<0.001
N0	36 (92.3)	3 (42.9)	4 (5.8)	3 (15.0)	
N1	3 (7.7)	4 (57.1)	65 (94.2)	17 (85.0)	
Distant metastasis					0.001
M0	39 (100.0)	5 (71.4)	60 (87.0)	14 (70.0)	
M1	0 (0.0)	2 (28.6)	9 (13.0)	6 (30.0)	
TNM stage					<0.001
I/II	36 (92.3)	3 (42.9)	4 (5.8)	2 (10.0)	
III/IV	3 (7.7)	4 (57.1)	65 (94.2)	18 (90.0)	
Vascular invasion	0 (0.0)	2 (28.6)	33 (47.8)	8 (40.0)	<0.001
Immunohistochemical features					
CgA (+)	32 (82.1)	4 (57.1)	57 (82.6)	15 (75.0)	0.367
Syn (+)	29 (74.4)	5 (71.4)	53 (76.8)	13 (65.0)	0.728
NSE (+)	9 (23.1)	2 (28.6)	16 (23.2)	2 (10.0)	0.594
CD56 (+)	16 (41.0)	4 (57.1)	38 (55.1)	5 (25.0)	0.086

*G-NENs* gastric neuroendocrine neoplasms, *NET G1* neuroendocrine tumor G1, *NET G2* neuroendocrine tumor G2, *NEC G3* neuroendocrine carcinoma G3, *MANEC* mixed adenoneuroendocrine carcinoma, *TNM* tumor-node-metastasis, *CgA* Chromogranin A, *Syn* synaptophysin, *NSE* neuron specific enolase

### Survival outcomes and prognostic factors

**Fig. 1:**
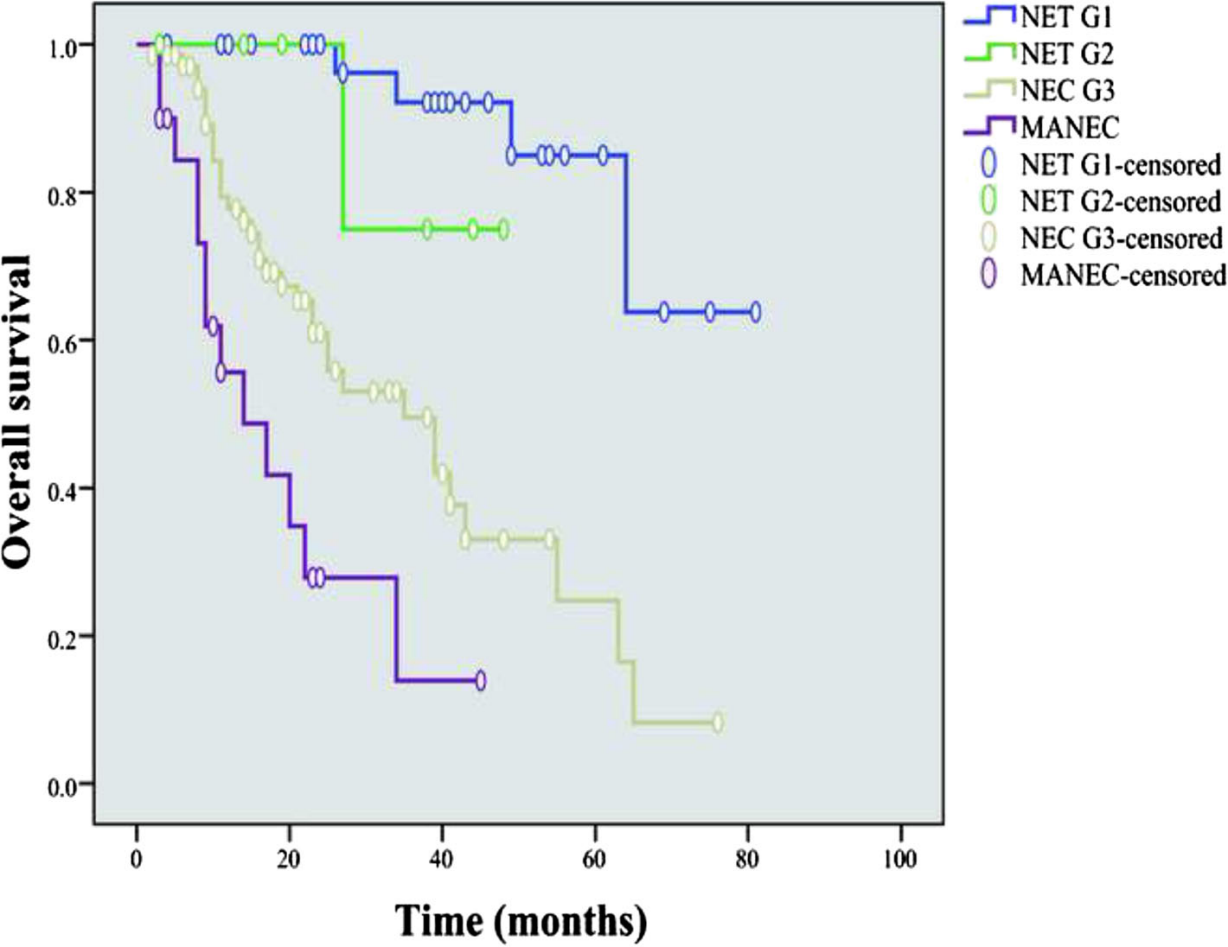
Overall survival of 114 G-NENs patients after radical resection (stratified by the new 2010 WHO classification). The NET G1 showed significant better OS compared with that of NEC G3 and MANEC (*P* < 0.001, and *P* < 0.001, respectively), but did not differ between NET G1 and NET G2 (*P* = 0.162), as well as those between NEC G3 and MANEC (*P* = 0.102)

**Fig. 2:**
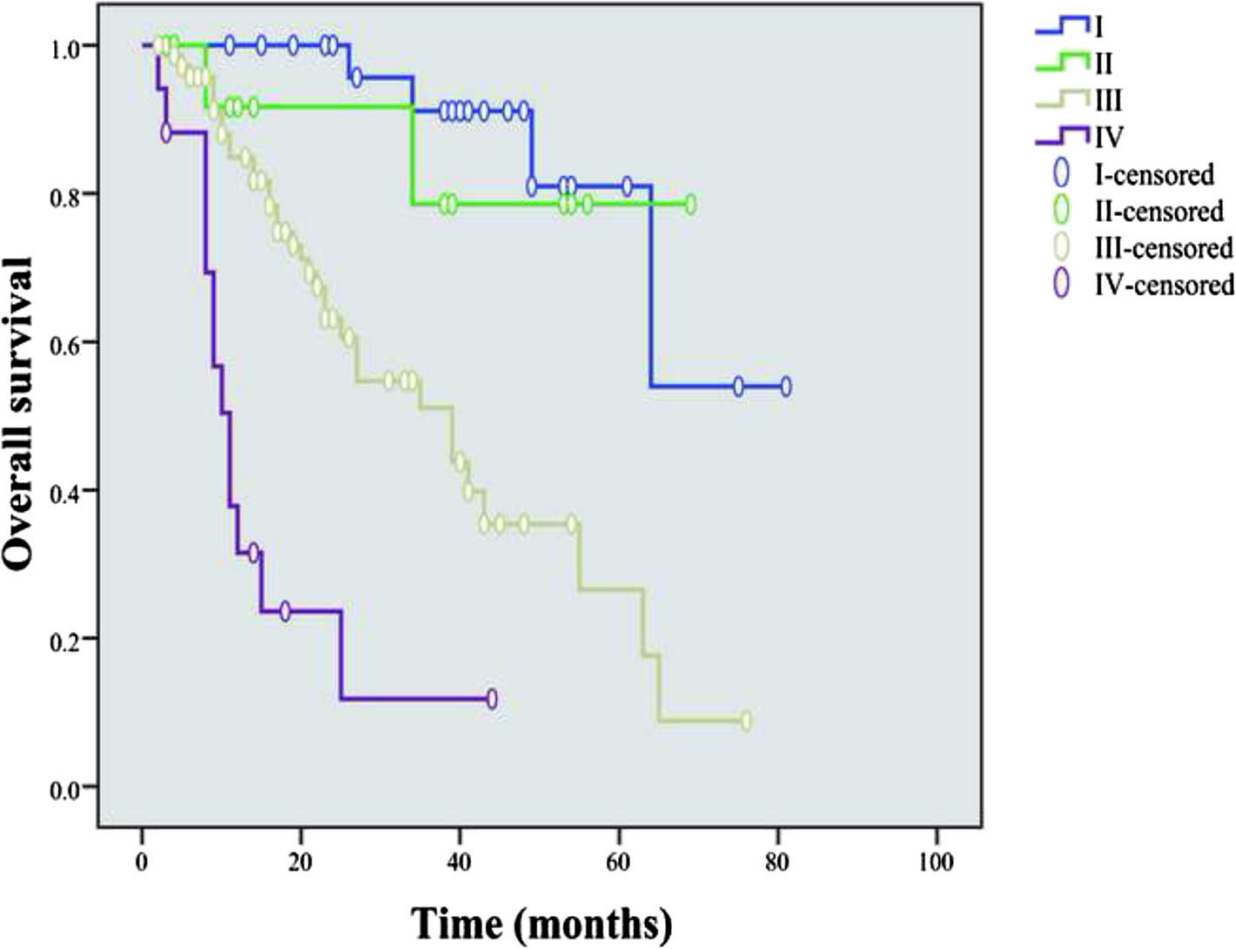
Comparison of overall survival in all patients with G-NENs of different TNM stages. The subgroups of patients with stage I and II obtained better OS than those in stage III and IV, respectively (I vs III, *P* < 0.001; I vs IV, *P* < 0.001; II vs III, *P* = 0.036; II vs IV, *P* < 0.001), as well as that between stage III and IV (*P* < 0.001), while no notable differences were found between stage I and II (*P* = 0.692)

**Fig. 3:**
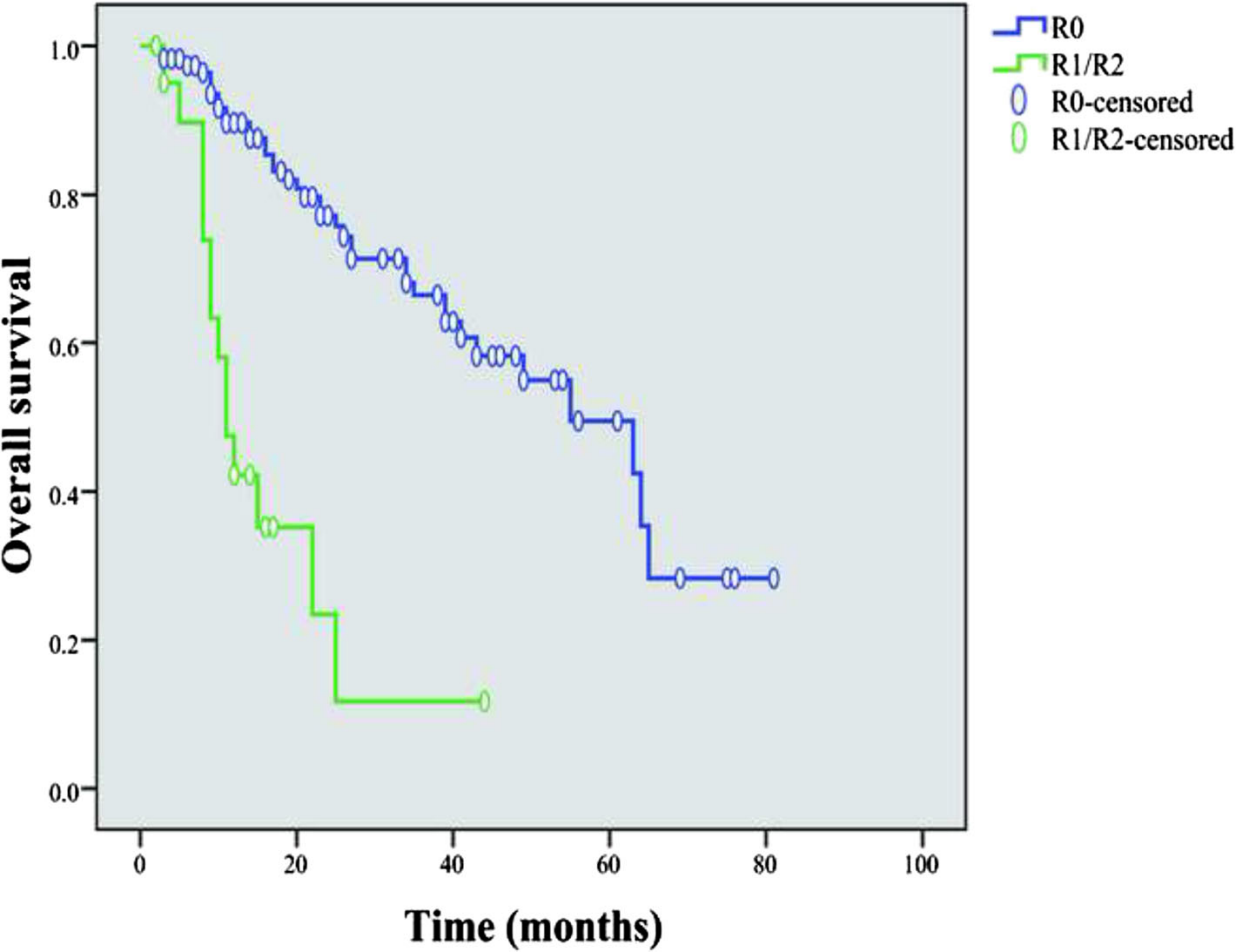
Comparison of survival in all patients with G-NENs of different surgical margins

With a median follow-up duration of 22 months (range, 2-81months), 52 patients died for the entire cohort. The main causes of the death were tumor related (76.9 %) and others (23.1 %, including respiratory failure, decompensated cirrhosis, stroke, and lung cancer). The OS rate for the entire cohort was 82.4 %, 59.0 % and 44.2 % at 1, 3 and 5 years, respectively. The 3-year OS for endoscopic resection was 92.9 %, while 51.4 % for open/laparoscopic surgery (*P* = 0.001). No significant difference was observed in survival between patients with and without chemotherapy (*P* = 0.758), as well as those between small cell and large cell type (*P* = 0.933). The subgroup patients with more advanced disease (NEC and MANEC) showed that these patients with chemotherapy had no survival benefit in comparison to those without chemotherapy (*P* = 0.730). The median survival was not achieved for both NET G1 and NET G2 versus 22.5 months (range 2–76) for NEC G3 and 12.5 months (range 3–45) for MANEC in patients with R0 resection. In those patients who underwent R0 resection, the NET G1 showed significant better OS compared with that of NEC G3 and MANEC (*P* < 0.001, and *P* < 0.001, respectively), but did not differ between NET G1 and NET G2 (*P* = 0.162, Fig. 1), as well as those between NEC G3 and MANEC (*P* = 0.102). The 3-year survival rate for the patients stratified by TNM stages I, II, III, and IV were 91.1 %, 78.6 %, 51.1 % and 11.8 %, respectively (*P* < 0.001, Fig. 2). The subgroups of patients with stage I and II obtained better OS than those in stage III and IV, respectively (I vs III, *P* < 0.001; I vs IV, *P* < 0.001; II vs III, *P* = 0.036; II vs IV, *P* < 0.001), as well as that between stage III and IV (*P* < 0.001), while no notable differences were found between stage I and II (*P* = 0.692). Moreover, we have found that patients who underwent R0 resection had better OS than that of R1/R2 resection (*P* < 0.001, Fig. 3), as well as females had greater prognosis than males (*P* = 0.029). OS was significantly greater in patients with lesion diameter ≤4 cm, NLR ≤2.8, and number of positive lymph node ≤4 (lesion diameter ≤4 cm vs >4 cm, *P* < 0.001; NLR ≤2.8 vs >2.8, *P* = 0.011; number of positive lymph node ≤4 vs >4, *P* < 0.001). The types of surgery, depth of invasion, lymph node metastasis, number of positive lymph node, distant metastasis, TNM stage, co-morbidity, surgical margin and the new 2010 WHO classification were significant factors of the prognosis for patients with G-NENs in the univariate analysis (*P* < 0.05). When coming into the multivariate analysis, only surgical margin and the new 2010 WHO classification were significant. The univariate and multivariate analyses by Cox regression model are listed in Table 4.

**Table 4:** Univariate and multivariate analysis of factors with os using cox proportional hazards regression modeling

Variables	Survival time, months	*P* univariate	*P* multivariate	Odds ratio* (95 % CI)
Gender				
Male	24.8 ± 18.6			
Female	29.4 ± 17.4	0.202		
Age, y				
≤ 60	26.5 ± 19.4			
> 60	25.6 ± 17.1	0.759		
Tumor marker				
Normal	26.7 ± 18.1			
Abnormal	23.9 ± 19.2	0.479		
NLR				
≤ 2.8	27.2 ± 18.4			
> 2.8	24.1 ± 18.7	0.340		
Chemotherapy				
Yes	26.4 ± 18.6			
N0	24.0 ± 16.9	0.576		
Types of surgery				
Endoscopic	66.0 ± 5.7			
Open/Laparoscopic	41.7 ± 3.4	0.001	0.580	0.6 (0.1 ~ 4.2)
Depth of invasion				
T1/T2	34.2 ± 19.5			
T3/T4	20.8 ± 15.5	<0.001	0.066	2.6 (0.9 ~ 7.2)
Lymph node metastasis				
N0	34.4 ± 20.4			
N1	21.8 ± 15.6	<0.001	0.277	0.3 (0.0 ~ 2.5)
Number of positive LN				
≤ 4	31.8 ± 20.5			
> 4	19.9 ± 13.2	<0.001	0.795	0.9 (0.4 ~ 2.0)
Distant metastasis				
M0	27.2 ± 18.4			
M1	12.9 ± 11.6	0.012	0.497	1.4 (0.5 ~ 4.1)
TNM stage				
I/II	35.0 ± 20.4			
III/IV	21.5 ± 15.3	<0.001	0.248	3.9 (0.4 ~ 38.5)
Lesions diameter				
≤ 4 cm	30.7 ± 19.2			
> 4 cm	20.1 ± 15.3	0.001	0.730	1.1 (0.5 ~ 2.4)
Co-morbidity*				
Absent	27.9 ± 18.3			
Present	19.7 ± 17.0	0.030	0.060	2.0 (0.9 ~ 4.1)
Surgical margin				
R0	28.6 ± 18.5			
R1/R2	12.6 ± 9.3	<0.001	0.002	3.9 (1.6 ~ 9.1)
WHO classification				
NET G1/G2	35.6 ± 197			
NET G3/MANEC	21.2 ± 15.5	<0.001	0.021	5.4 (1.3 ~ 22.9)

*OS* overall survival, *NLR* Neutrophil-Lymphocyte Ratio, *CI* confidence interval, *LN* lymph node, Co-morbidity* including diabetes mellitus, chronic pulmonary disease, cardiovascular, chronic liver and renal disease; *WHO* World Health Organization, *NET G1* neuroendocrine tumor G1, *NET G2* neuroendocrine tumor G2, *NEC G3* neuroendocrine carcinoma G3, *MANEC* mixed adenoneuroendocrine carcinoma

## Discussion

Although the annual incidence of G-NENs has been increased globally in recent decades, data on management of G-NENs has been poorly described, due to their rarity and with a spectrum of biological behaviors from benign to malignant. This study has provided comprehensive information on the clinicopathological characteristics, surgical outcome and prognosis of G-NENs with a relative large sample in a single institution. Moreover, we here discuss their prognostic predictors. Our results suggested that there was an obvious preponderance of males and G-NENs with high-grade (NEC G3 and MANEC) for this cohort. OS was significantly greater in patients with lesion diameter ≤4 cm, NLR ≤2.8, number of positive lymph node ≤4, females and R0 resection. Moreover, surgical margin and the newly updated WHO classification have been demonstrated to be closely associated with OS for G-NENs.

As described previously, for NENs, the male-to-female ratio ranged from 0.7 ~ 1.2 [7, 15, 16], while our results showed that a male-to-female ration for this cohort was 2.6. This discrepancy in organ distribution and sex ration may suggest ethnic differences in the development of NENs. Thus, it is of utmost importance to carry out further clinical epidemiology researches with bigger sample and multicentre in China. Distant metastases can be detectable at the time of diagnosis in 12.9 % of patients with neuroendocrine tumors [17]. In this study, a total of 12.6 % patients with G-NENs who underwent surgery had distant metastasis. Apart from the regional lymph nodes, the hepatic metastasis was the most common for neuroendocrine tumors [18], which was in accordance with ours finding. Generally, NENs were more indolent than carcinomas, and the symptoms of NENs were nonspecific for many cases [7, 8]; but abdominal pain (51.4 %) was the most important clinical manifestations [19]. In our study, 84 patients (62.2 %) exhibited abdominal pain/discomfort, and 18 patients (13.3 %) were asymptomatic.

In order to improve the prognostic classification of the NENs and to better guide therapeutic strategies, the WHO updated its classification system in 2010 and divided G-NENs into NET G1, NET G2, NEC G3 and MANEC. However, this scheme may be problematic for classifying NENs on small bioptic specimens due to tissue may not be sufficient to evaluate Ki-67 index and count mitoses, and become a major concern for pathologists sometimes. NET G1 predominated in the gastrointestinal tract (stomach, rectum, and small intestine) [20, 21]; but our data suggested that NEC G3 predominated for G-NENs. The NEC G3 was more frequent in males (male/female ration of 2) with an average age of 65 years and usually located in the cardia [1, 22]. In the present study, 52.2 % patients with NEC G3 arose in the upper third of stomach, and male-dominated with a mean age of 61 years, which was similar to their results. As described previously, the pancreatic NET G2, NEC G3 and MANEC often had lymph node invasion [23], as well as distant metastasis. Similarly, we have found that lymph node metastasis occurred significant more in tumors with higher-grade. Furthermore, patients with NET G1 had less cases with vascular invasion than that of NET G2, and NEC G3 and MANEC (*P* = 0.032, *P* < 0.001, and *P* < 0.001, respectively) in this study. Of note, NET G1 was mostly in stage I /II (92.3 %); but those with NET G2, NEC G3 and MANEC were mainly in stage III/IV (57.1 %, 94.2 %, and 90.0 %, respectively).

Nowadays, surgery should be the initial treatment if the G-NENs are technically resectable, and with a basic principle of radical resection without considering grade and stage [24]. Previous study indicated that patients with NENs who underwent R0 resection obtained a statistically longer survival than those with R1/R2 resection [25], which was similar to ours results. Patients with palliative resection may relieve discomfort from the size of the metastases/tumors or endocrine symptoms. In some unresectable NENs, palliative resection of liver metastases had a longer survival and better symptoms relief than non-surgical treatment, as previously reported [26]. Partelli et al. reported that patients with liver metastases benefit from palliative resection when compare to those who were conservatively treated (median OS: 89 vs 36 months, *P* < 0.05) [27]. However, whether palliative removal of the primary tumor or distant metastases can really results in a survival benefit for G-NENs still remains controversial. Thus, further clinical trial comparing non-surgical management to palliative resection of tumor/metastases for unresectable G-NENs should be performed. The surgical approaches differed from tumor location and size. ER to treat foregut NET has been increasingly preformed in recent years. Jung et al. has demonstrated that ER of foregut NENs can be safely performed in selected cases (tumor size < 20 mm, as well as lesions confined to the submucosal layer) [5]. In our study, the tumor size was 0.7 ± 0.4 cm for ER, and the depth of invasion was mostly T1 for ER (24/25 vs 15/110 for open/laparoscopic surgery). G-NENs who underwent ER had a good prognosis with a 3-year OS of 92.9 %. By contrast, G-NENs with open/laparoscopic surgery had a greater tumor size (4.5 ± 2.3 cm), and with a more aggressive clinical course. Systemic chemotherapy may be used for non-pancreatic NENs and G-NENs of high-grade or aggressive clinical course [6, 28]. Chemotherapy had improved the median survival for G-NENs patients with more advanced disease [29]. However, the subgroup patients with more advanced disease (NEC and MANEC) showed that these patients with chemotherapy had no survival benefit in comparison to those without chemotherapy in the present study (*P* = 0.730). This phenomenon could be attributed to the fact that some patients received different chemotherapy cycles and regimens; what’s more, only a small number of patients with NEC and MANEC received chemotherapy in this study. To date, there were no conclusions regarding which regimen was the most effective due to rarity of G-NENS. Therefore, prospective, multicenter, randomized clinical trials to verify the efficacy of chemotherapy for G-NENs are still warranted.

To date, limited data concerning prognoses of patients with primary G-NENs are available, especially stratified by new 2010 WHO classification. Overall prognosis was favorable for NENs, and 5-year survival rate ranged from 75 to 85 % [7, 20]. In the present study, patients had an unfavorable prognosis, with a 5-year OS of 44.2 %. This phenomenon could be attributed to the fact that the research consisted of a high proportion of NEC G3 and MANEC (65.9 %) in this study. In addition, the patients with malignant NENs had a worse prognosis, with 5-year survival of 45.9–50.4 % [25, 30]. We have also reported that the median survival was not achieved for both NET G1 and NET G2 versus 22.5 months (range 2–76) for NEC G3 and 12.5 months (range 3–45) for MANEC in patients with R0 resection. Survival was significant greater in women, which was in agreement with previous study [7]. Additionally, there was no significant difference in OS between large cell and small cell type for G-NENs, which was similar to other report [31]. For the NENs, several literatures mentioned that tumor grade, distant metastasis, TNM stage, tumor location and size, and age were independent predictors for outcome in univariate or multivariate analysis [7, 23, 32]. In the present cohort of primary G-NENs, types of surgery, depth of invasion, lymph node metastasis, number of positive lymph node, distant metastasis, TNM stage, co-morbidity, surgical margin and the new 2010 WHO classification were significant factors of the prognosis for patients with G-NENs in the univariate analysis (*P* < 0.05). However, surgical margin and the new 2010 WHO classification remained the only independent indicators for prognosis in the multivariate analysis. It was worth noting that the new 2010 WHO classification may not woke well in classifying NET G1 and NET G2 into different prognostic categories in the present study, with a similar conclusion by Yang et al. [33] and Kim et al. [31]. As such, further studies are still needed to evaluate the prognostic value of the new WHO criteria based on large populations. TNM stage I and II cross on the Kaplan-Meier curve at 5 years may be relevant to its’ low-efficacy to stratify G-NENs into different prognostic group, as well as due to the small number of patients diagnosed with stage I and II in this study. The factors such as gender, depth of invasion, lymph node metastasis, and TNM stages etc., may somehow affect prognosis, but could not be an independent prognostic factor with much significance for G-NENs in comparison to surgical margin and the new 2010 WHO classification.

However, our study had some limitations. Firstly, the findings should be carefully interpreted due to the small number of patients and retrospective nature. Furthermore, we have demonstrated that 2010-WHO classification works well in stratifying G-NENs into different prognostic categories; but this scheme is merely a histological classification, which may be not sufficient to predict tumor progression. Thus, further studies are needed to develop optimal staging system to use. To date, the optimal medication regimens for gastric NENs have not well been established yet, including chemotherapy and/or targeted therapy. Unfortunately, our data cannot provide enough evidence to support any notions now. Therefore, further explorations should be carried out, and we believe that this can bring the benefit of comprehensive treatment for gastric NENs to clinical practice in the future.

## Conclusions

In sum, G-NENs are a kind of rare tumors, and patients with NET G3 and MANEC have an unfavorable prognosis even surgically treated. Moreover, we have demonstrated that the new 2010 WHO classification might be a valuable tool to stratify G-NENs into different prognostic categories, combining with surgical margin were meaningful prognostic factors of G-NENs.

## Abbreviations

A/G: Albumin/globulin
CT: Computerized tomography
ENETS: European Neuroendocrine Tumor Society
ER: Endoscopic resection
G-NENs: Gastric neuroendocrine neoplasms
MANEC: Mixed adenoneuroendocrine carcinoma
NEC G3: Neuroendocrine carcinoma G3
NET G1: Neuroendocrine tumor G1
NET G2: Neuroendocrine tumor G2
NLR: Neutrophil-lymphocyte ratio
OS: Overall survival
RGETNE: Registry for Gastroenteropancreatic Neuroendocrine Tumors
SEER: Surveillance, Epidemiology, and End Results Program
TNM: Tumor-node-metastasis
UICC/AJCC: Union for International Cancer Control and the American Joint Cancer Committee
WHO: World Health Organization
